# BioMeQ-MD: Developing Biomechanical Interventions for Major Depression

**DOI:** 10.1192/bjo.2025.10105

**Published:** 2025-06-20

**Authors:** Paul M Briley, Sean Lowton-Smith, Callum Osler, Tom Outram, Simon Briley

**Affiliations:** ^1^Faculty of Medicine and Health Sciences, University of Nottingham, Nottingham, United Kingdom; ^2^School of Sport and Exercise Science, University of Derby, Derby, United Kingdom

## Abstract

**Aims:** Changes in body biomechanics – that is, changes in gait, posture, and balance – have been identified during episodes of major depressive disorder (MDD). Whilst biomechanical changes are related to the level of disability experienced by people with MDD, such changes are rarely asked about by clinicians or addressed directly by interventions. As part of a project studying whether interventions that target biomechanics might be helpful for MDD, we are conducting an initial non-patient study quantifying biomechanics, mood and anxiety, before and after physiotherapist-directed interventions.

**Methods:** Twenty young people (aged 19–21) from a higher education setting have completed baseline measurements so far. The baseline protocol consists of questionnaire measures, a happy–sad emotional bias task, and a comprehensive biomechanical assessment including walking tasks, static and dynamic balance tasks, and postural measures.

The baseline walking-task metrics described here were collected using a force plate flush with the floor of a 10-metre walkway. Participants were asked to walk at a comfortable pace across the walkway six times. Walking speed was recorded, and the plate measured reaction forces from which were derived peak/mean forces in three dimensions, as well as variability in these forces across the six repetitions. There were no exclusion criteria for baseline analyses, other than physical disability preventing completion of key measures.

**Results:** In the fifteen participants (13 male) for whom baseline analyses are complete, mean PHQ-8 (self-reported depressive symptoms) ranged from 0–11 (none to low-moderate), and mean GAD-7 (self-reported anxiety) ranged from 0–25 (none to severe). One participant was taking sertraline, the others reported no mental health medications. Ratings of pain and mobility difficulties were low (EQ-5D domains, scored 1–5: fourteen participants scored 1, one participant scored 2).

Whilst there were no significant relationships between PHQ-8 total score and baseline gait metrics, mean reaction time to sad faces on the emotional bias task was correlated with *variability* in vertical and horizontal walking forces (28–50% variance explained, *r*^*2*^, across individual metrics, *p*=0.042–0.003). Greater GAD-7 total score was associated with greater walking speed and mean vertical and horizontal force (32–39% variance explained, *p*=0.029–0.012).

**Conclusion:** Gait variability was associated with a sensitive mood marker (average reaction time to sad faces), in this non-patient sample with low levels of self-reported depression. Self-reported anxiety was associated with average walking force and speed. Ongoing work is examining changes in metrics following physiotherapist-directed interventions and adapting the approach, with lived experience experts, for clinical studies.

